# Joint associations of objectively-measured sedentary behavior and physical activity with health-related quality of life

**DOI:** 10.1016/j.pmedr.2015.11.004

**Published:** 2015-11-14

**Authors:** Paul D. Loprinzi

**Affiliations:** Center for Health Behavior Research, Department of Health, Exercise Science, and Recreation Management, The University of Mississippi, University, MS, United States

**Keywords:** Epidemiology, Physical activity, Sedentary behavior, Quality of life

## Abstract

No studies, to my knowledge, have examined the joint effects of physical activity and sedentary behavior on health-related quality of life (HRQOL), which was the purpose of this study. Data from the 2003–2006 National Health and Nutrition Examination Survey (NHANES) were used (N = 5,536). Moderate-to-vigorous physical activity (MVPA) and sedentary behavior were assessed using an ActiGraph 7164 accelerometer, with HRQOL assessed using the Centers for Disease Control and Prevention 4-item HRQOL index. MVPA (β_adjusted_ = − 0.01; 95% CI: − 0.01 to − 0.004; P < 0.001), but not sedentary behavior (β_adjusted_ = − 0.0003; 95% CI: − 0.001–0.0001; P = 0.37), was associated with HRQOL. MVPA was associated with HRQOL among those above the median (≥ 487.5 min/day) level of sedentary behavior (β_adjusted_ = − 0.02; 95% CI: − 0.03 to − 0.01; P = 0.006; N = 2769). The results of this brief report do not demonstrate that sedentary behavior, independent of MVPA, is associated with HRQOL. The independent association of MVPA on HRQOL confirms previous work that used self-report measures of MVPA.

## Highlights

• National sample employed
• Joint effects of sedentary and physical activity and quality of life employed
• No joint effect was observed.
• Sedentary behavior was not associated with perceived quality of life.
• Physical activity was independently associated with quality of life.

## Introduction

Emerging research suggests that sedentary behavior is associated with poor cardiometabolic health outcomes, independent of participation in moderate-to-vigorous physical activity (MVPA) (de Rezende et al., 2014). Fewer studies, however, have examined the “joint” effects of sedentary behavior and MVPA on health. In a recent study employing an objective measure of MVPA and sedentary behavior, Schmid et al. (Schmid et al., 2015) demonstrated independent associations of sedentary behavior and MVPA with mortality, but sedentary behavior was not associated with mortality among those engaging in greater levels of MVPA, which is in alignment with a recent meta-analysis (Biswas et al., 2015). Although studies have examined and demonstrated a favorable association between self-reported MVPA and health-related quality of life (HRQOL) (Klavestrand and Vingard, 2009, Heath and Brown, 2009, Bize et al., 2007, Freelove-Charton et al., 2007, Kekkonen et al., 2007, Kruger et al., 2007, Mukamal et al., 2006, Pierce et al., 2006, Abell et al., 2005, Smith and McFall, 2005), examination of any potential joint effects of objectively-measured sedentary behavior and MVPA on HRQOL has yet to be explored, which was the purpose of this brief report. I examine this association in the broader adult population using data from the population-based National Health and Nutrition Examination Survey (NHANES). Here, HRQOL is conceptualized as an individual's perceived physical and mental health.

## Methods

### Study design and participants

Data from the population-based 2003–2006 NHANES were used. The 2003–2006 cycles were used because these are the only present NHANES cycles with accelerometer data. Briefly, NHANES employs a population-based sample of Americans via household interviews and examinations in a mobile examination center. Using a multistage, complex probability design, non-institutionalized U.S. civilians are selected for participation. The design consists of 4 stages, including the identification of counties, segments (city blocks), random selection of households within the segments, and random selection of individuals within the households.

In the 2003–2006 NHANES cycles, 10,020 adults (20 + yr) were enrolled. After excluding those with missing covariate data, 8804 remained. After excluding those with missing or insufficient accelerometry data, 5848 remained. Lastly, after excluding those with missing HRQOL data, 5536 remained, which constituted the analytic sample. Table 1 displays the weighted characteristics of the study variables among the analyzed sample.

### Measurement of MVPA and sedentary behavior

MVPA and sedentary behavior were assessed using the ActiGraph 7164 accelerometer. Consistent with other studies (Loprinzi, 2015a, Loprinzi, 2015b, Troiano et al., 2008), SAS (version 9.2) was used to reduce accelerometry data to those with ≥ 4 days of ≥ 10 h/day of monitored data and integrate it into 1 min time intervals. Nonwear time was identified as ≥ 60 consecutive minutes of zero activity counts, with allowance for 1–2 min of activity counts between 0 and 100. The Troiano cut-point (2020 counts/min) was used to determine time spent in MVPA (Troiano et al., 2008). Sedentary behavior was defined as counts/min ≤ 99 (Matthews et al., 2008). Using these cut-points, total daily estimates of sedentary behavior and MVPA were calculated.

### Measurement of HRQOL

The Centers for Disease Control and Prevention HRQOL measure was assessed from 4 questions, including 1 question about self-rated health status and 3 about the number of unhealthy days during the past 30 days. The HRQOL-4 developed by the Centers for Disease Control and Prevention has undergone extensive reliability and validity testing and has demonstrated adequate psychometric properties (Jiang and Hesser, 2009, Horner-Johnson et al., 2009, Hays et al., 2009, Mielenz et al., 2006, Linden et al., 2005). The 4 items include:

1. “Would you say that in general your health is excellent, very good, good, fair, or poor?”
2. “Now thinking about your physical health, which includes physical illness and injury, how many days during the past 30 days was your physical health not good?”
3. “Now thinking about your mental health, which includes stress, depression, and problems with emotions, how many days during the past 30 days was your mental health not good?”
4. “During the past 30 days, approximately how many days did poor physical or mental health keep you from doing usual activities, such as self-care, work, or recreation?”

The 4 CDC HRQOL items were categorized according to CDC's recommendations (Horner-Johnson et al., 2009, Hays et al., 2009), which included question 1 dichotomized as good/excellent health (coded as 0) or poor/fair health (coded as 1). The latter 3 items were dichotomized as 14 or more days (coded as 1) and less than 14 days (coded as 0).

Thus, the recoded 4 HRQOL items ranged from 0–1. An overall HRQOL score was created by summing the responses from each of the 4 individual items, with higher scores indicating worse HRQOL (range: 0–4).

### Analysis

Statistical analyses were performed via procedures from survey data using Stata (v. 12). To account for oversampling, non-response, non-coverage, and to provide nationally representative estimates, all analyses included the use of survey sample weights, clustering and primary sampling units. In particular, recalculated sample weights to account for missing/insufficient accelerometer data were applied to minimize bias associated with accelerometer non-compliance. Multivariable ordinal regression was employed to examine the association between MVPA/sedentary behavior with HRQOL. In all models, covariates included age, gender, race-ethnicity, self-reported smoking status (current vs. not), measured body mass index (kg/m^2^; continuous), education (college or more vs. less), C-reactive protein (mg/dL; continuous) and physician-diagnosis of coronary artery disease (yes vs. no). Age, gender, race-ethnicity, education and coronary artery disease status were self-reported. Body mass index was measured using standard procedures. Blood samples were obtained to assess high sensitivity C-reactive protein, using latex-enhanced nephelometry.

Similar to other studies (Schmid et al., 2015), I used the median-split method for the effect modification analyses.

Five multivariable ordinal regression analyses were computed:

Model 1) Examining the independent association of MVPA and sedentary behavior on HRQOL; MVPA (continuous), sedentary behavior (continuous) and the covariates were included in the model.
Model 2) A multiplicative interaction model was computed by creating a cross-product term (sedentary behavior ∗ MVPA) along with their main effects (and the covariates) in a multivariable ordinal regression model.
Model 3) Examining the association between MVPA and HRQOL among those above the median level (consistent with other studies (Schmid et al., 2015)) of sedentary behavior (i.e., ≥ 487.5 min/day); MVPA (continuous) and the covariates were included in the model, with the model restricted to those above the median level of sedentary behavior.
Models 4–5) Examining the association between sedentary behavior and HRQOL among those above (i.e., ≥ 14 min/day) and below (i.e., < 14 min/day) the median level of MVPA.

## Results

Unadjusted sedentary behavior levels among those with a HRQOL score of 0, 1, 2, 3 and 4, respectively, were 483.2 min/day, 480.6 min/day, 487.2 min/day, 518.2 min/day, and 523.5 min/day. Unadjusted MVPA levels across these 5 groups were 25.9 min/day, 20.4 min/day, 17.6 min/day, 9.7 min/day and 9.8 min/day.

### Model 1 (independent association)

MVPA (β_adjusted_ = − 0.01; 95% CI: − 0.01 to − 0.004; P < 0.001), but not sedentary behavior (β_adjusted_ = − 0.0003; 95% CI: − 0.001–0.0001; P = 0.37), was associated with HRQOL. As stated previously, a higher HRQOL score indicates poor HRQOL, so this finding indicates that greater MVPA was associated with better HRQOL.

### Model 2 (multiplicative interaction)

The multiplicative interaction term for sedentary behavior and MVPA on HRQOL approached significance (β_adjusted_ = − 0.00003; P = 0.08).

### Model 3 (MVPA on HRQOL among those above the 50th percentile for sedentary behavior)

MVPA was associated with HRQOL among those above the median sedentary behavior (β_adjusted_ = − 0.02; 95% CI: − 0.03 to − 0.01; P = 0.006; N = 2769).

### Models 4–5 (sedentary behavior on HRQOL among those above and below the 50th percentile for MVPA)

Sedentary behavior was not associated with HRQOL among those above the median MVPA (β_adjusted_ = − 0.0004; 95% CI: − 0.001–0.0007; P = 0.46; N = 2739). Similarly, sedentary behavior was not associated with HRQOL among those below the median MVPA (β_adjusted_ = − 0.0001; 95% CI: − 0.001–0.0009; P = 0.84; N = 2797).

## Discussion

The results of this brief report do not demonstrate that sedentary behavior, independent of MVPA, is associated with HRQOL. The independent association of MVPA on HRQOL confirms previous work that used self-report measures of MVPA (Klavestrand and Vingard, 2009, Heath and Brown, 2009, Bize et al., 2007, Freelove-Charton et al., 2007, Kekkonen et al., 2007, Kruger et al., 2007, Mukamal et al., 2006, Pierce et al., 2006, Abell et al., 2005, Smith and McFall, 2005), as well as a study utilizing an objective measure of MVPA (Phillips et al., 2015). The potential disconnect between the association of sedentary behavior and cardiometabolic parameters vs. sedentary behavior and HRQOL, warrants future work. Although speculative, it may be reasonable to think that prolonged sedentary behavior may induce cardiometabolic consequences (e.g., modulation of inflammation and/or increases in the enzyme lipoprotein lipase, which favors increased accumulation of triglycerides) (Hamilton et al., 2007, Tremblay et al., 2010), but then does not have a negative influence on perceived quality of life, especially if the individual is able to engage in MVPA. For example, even if an individual sits for the majority of their day, but still engages in structured MVPA, they may, despite possibly having compromised cardiometabolic parameters, still perceive their physical and mental health as adequate given their potential perceived benefits of engaging in structured MVPA.

In conclusion, sedentary behavior was not independently associated with HRQOL and did not moderate the association between MVPA and HRQOL. The independent association of MVPA on HRQOL is an important finding, as HRQOL is linked with prolonged survival (Landman et al., 2010). The present findings underscore the perceived physical and mental health benefits associated with MVPA. Future prospective work, however, is needed to confirm the present findings.

## Conflicts of interest

No funding was used to prepare this manuscript and all authors disclose no conflicts of interest.

## Figures and Tables

**Table 1:** Characteristics of the analyzed sample, 2003–2006 NHANES (N = 5536).

Variable	Point estimate	95% CI
Age, mean yr	46.6	45.6–47.9
Female, %	51.5	50.0–52.9
Non-Hispanic white, %	73.5	69.2–77.9
Some college or more, %	59.6	56.8–62.4
Smoker, %	21.1	19.5–22.8
Coronary artery disease, %	3.4	2.8–4.1
CRP, mg/dL	0.40	0.37–0.43
Body mass index, kg/m^2^	28.2	27.9–28.6
MVPA, mean min/day	24.0	22.8–25.2
Sedentary, mean min/day	484.1	480.4–487.9
HRQOL, mean	0.37	0.34–0.41
HRQOL, %		
0 (n = 3820)	74.9	72.9–76.8
1 (n = 1142)	16.5	15.1–17.9
2 (n = 362)	5.3	4.6–5.9
3 (n = 159)	2.4	1.7–3.0
4 (n = 53)	0.9	0.6–1.1

CI, Confidence Interval.

CRP, C-reactive protein.

HRQOL, health-related quality of life.

MVPA, moderate-to-vigorous physical activity.

NHANES, National Health and Nutrition Examination Survey.
